# Development of a recombinant protein based ELISA for the diagnosis of canine leptospirosis

**DOI:** 10.1186/1753-6561-8-S4-P139

**Published:** 2014-10-01

**Authors:** Wallace Pereira, André Alex Grassmann, Micaela Domingues, Flávia McBride, Alan McBride

**Affiliations:** 1 Núcleo de Biotecnologia, CDTec, Universidade Federal de Pelotas, Pelotas, Brazil; 2 Medicina, Universidade Católica de Pelotas, Pelotas, Brazil

## Background

Leptospirosis is an emergent zoonotic disease caused by pathogenic *Leptospira *spp., and humans are regarded as an accidental or dead-end host [1]. The global incidence of severe human leptospirosis was estimated to be approximately 500.000 cases worldwide, although this is likely an underestimate due to the lack of a reliable diagnostic test. Mortality ranges from 10 to > 50% for Weil's disease or severe pulmonary hemorrhage syndrome (SPHS), respectively [2]. The microscopic agglutination test (MAT) is the standard test for this disease, however, it requires paired serum samples, is laborious, difficult to analyze and subjective [3]. Apart from the danger connected with rodents, which are the main vectors of leptospires, occurrence of the disease in dogs can generate a higher risk of infection for humans. Thus, a novel sensitive and specific serological test providing a rapid and secure diagnosis is urgently required for the laboratorial diagnosis of leptospirosis. In this study, 15 recombinant proteins from *L. interrogans *were expressed, purified and evaluated in an indirect ELISA using canine sera that were characterized by the MAT and a whole-cell *Leptospira *ELISA.

## Methods

Fifteen recombinant polypeptides based on LigB, LigA LipL32, LemA, OmpL37, FlaA1 and FlaB1 were cloned and expressed in *Escherichia coli*, purified by Ni^2+^affinity chromatography, quantified and stored at -20 ºC until use. The recombinant proteins were assessed in a checkerboard ELISA to evaluate their capability discriminate between infected and non-infected canine sera, and to determine the optimal combinations of antigen, canine sera and anti-dog IgG and IgM HRP conjugate antibodies. After the initial screening, two recombinant proteins were selected and tested against a serum panel, 10 positive and 10 negative serum samples.

## Results and conclusions

Following the preliminary screening using pooled sera, rLigBrep and rLipL32 were evaluated using a panel of individual canine serum samples. The optimal concentrations of antigen for the coating step was 100 ng/well. The optimal sera dilution was 1:200 for rLigBrep, 1:100 for rLipL32, 1:2500 for the IgG and 1:5000 for the IgM conjugates. Based on the negative sera, the average OD for rLigBrep was 0.24 ± 0.11 for the negative pool and the cut-off was calculated as OD = 0.47. 100% of the negative sera were negative and 70% of the characterized positive were identified as positive under these conditions. The average OD based on rLipL32 was 0.2 ± 0.08 for the negative pool, equivalent to a cut-off of OD = 0.36. 100% of negative sera were negative and 50% positive sera were identified as positive. The anti-dog-IgG conjugate exhibited the best specificity and sensitivity. The recombinant protein rLigBrep demonstrated potential as a diagnostic tool for canine leptospirosis, 70.0% sensitivity and 100% specificity. The native protein, LigB, is highly conserved in pathogenic *Leptospira *spp. and therefore represents an ideal candidate for further studies.
